# Detection of interaction articles and experimental methods in biomedical literature

**DOI:** 10.1186/1471-2105-12-S8-S13

**Published:** 2011-10-03

**Authors:** Gerold Schneider, Simon Clematide, Fabio Rinaldi

**Affiliations:** 1 Institute of Computational Linguistics, University of Zurich, 8050 Zurich, Switzerland

## Abstract

**Background:**

This article describes the approaches taken by the OntoGene group at the University of Zurich in dealing with two tasks of the BioCreative III competition: classification of articles which contain curatable protein-protein interactions (PPI-ACT) and extraction of experimental methods (PPI-IMT).

**Results:**

Two main achievements are described in this paper: (a) a system for document classification which crucially relies on the results of an advanced pipeline of natural language processing tools; (b) a system which is capable of detecting all experimental methods mentioned in scientific literature, and listing them with a competitive ranking (AUC iP/R > 0.5).

**Conclusions:**

The results of the BioCreative III shared evaluation clearly demonstrate that significant progress has been achieved in the domain of biomedical text mining in the past few years. Our own contribution, together with the results of other participants, provides evidence that natural language processing techniques have become by now an integral part of advanced text mining approaches.

## Introduction

Results from genetic and biomedical research are published on a daily basis. They appear in scientific articles, accessible through online literature services like PubMed (http://pubmed.gov). More than 20 million citations are currently available through PubMed. One topic of great interest is protein-protein interactions, which play fundamental roles in biological processes (e.g. signal transduction). Biologists routinely perform experiments in order to detect or confirm protein interactions. In doing so, they use a variety of experimental methods. Databases such as IntAct [1], MINT [2] and BioGRID [3] aim at collecting the known interactions from the literature. The process of extracting selected items of information from the published literature in order to store such items in databases is known as *curation*. Manual curation is very costly and cannot keep up with the rate of data generation [4]. Tools that can support the process of curation are thus very useful for the community.

The detection of protein-protein interactions (PPIs) and experimental methods has been the scope of one of the tasks of the recent BioCreative III competitive evaluation of text mining systems. The subtask PPI-ACT is a binary classification task aiming at classifying biomedical research texts into the class of articles which contain descriptions of protein-protein interactions (called “curatable articles”) and the class of articles which do not contain such interactions. Such a classification can for example help curators as they do not need to read irrelevant articles. The subtask PPI-IMT can be seen as a named-entity recognition task aiming to deliver which experimental methods are used in a biomedical article. Alternatively, it can be seen as a multi-label document classification task, since participants are only required to deliver the methods mentioned in the article, and not the positions where they appear. Fully automated extraction of information from the literature is currently unrealistic, but text mining tools are already sufficiently reliable to provide hints to the curators, and have been shown to speed up their activities [5]. An interesting observation of [6] is that the curators, although in general considering the results from the NLP tool as helpful, clearly preferred a high recall setting to one chosen to optimize precision or F-score, because it is much easier and less time-consuming to reject suggestions (false positives, low precision) than to add new information from scratch (false negatives, low recall). However, a very low precision (i.e. an excessive number of false positives) is equally negative, as it was observed in the interactive task (IAT) of BioCreative III [7], because the curators would have to reject numerous suggestions by the system which (to the human expert) are obviously wrong.

We have participated in several previous shared tasks, in text mining tasks like protein-protein interaction [8,9] and method detection [10]. We had not earlier considered participation to the PPI-ACT task, because at first sight it appears to be a pure document classification task where an NLP-rich approach would not be able to provide a significant contribution. The participation to the PPI-ACT task of BioCreative III was motivated by the desire to dispel this negative assumption, by enriching a traditional machine learning (ML) approach with features derived from our PPI extraction pipeline.

## Methods

In this section, we describe the methods used in the task of experimental method detection and article classification.

### Article classification task (PPI-ACT)

We used a classical approach to the PPI-ACT task, based on a document classifier (in our case a maximum entropy classifier) making use of standard textual features, crucially enhanced with a feature derived from a pre-existing pipeline aimed at extracting protein-protein interactions [9,11].

The features which we adopted in the official task are derived from the sources listed below:

• words occurring in an article

• MeSH index terms (http://www.nlm.nih.gov/mesh) appearing as meta-information in an article

• results from running our full protein-protein interaction detection pipeline from the BioCreative II.5 challenge on an article

For all experiments, only the development set was used as training material for the test runs (as only the class distribution of the development set was declared to be representative for the test set by the BioCreative organizers). The following three feature groups were at work in our submissions (each feature is represented by a single letter which will be used in the rest of this article as a reference).

**Bag of word features (W)** All words of the articles were stemmed, then all counts of a stem were used as a feature. E.g, if the word “protein” was found 3 times, we generated the features “protein 1”, “protein 2”, “protein 3”.

**MeSH features (M)** Every MeSH descriptor, with and also without every qualifier, was turned into features. E.g., for the MeSH term “-Signal Transduction (-drug effects; +physiology)” as it appeared in the textual format, we produced the descriptor features “signal/transduction/drug/effects”, “signal/transduction/physiology”. For multi-word terms, we added also all descriptor terms produced by iteratively removing the first word, for instance “transduction”. Additionally, all MeSH qualifiers as “-drug/effects” and “+physiology” were added.

**PPIscore features (P)** This feature is computed using the full pipeline for detection of PPI as used in the BioCreative II.5 challenge [9,11]. It includes in particular the recognition of terms from UniProt [12], EntrezGene [13], methods section of the PSI-MI ontology (Proteomics Standard Initiative - Molecular Interactions) [14], and CLKB (Cell Line Knowledge Base) [15]. Further, chunking and full syntactic dependency parsing [16] is done for sentences containing more than one term. The original system is used to detect candidate interactions, and delivers each of them, together with a numerical score. This value, which will be referred to as “PPIscore” in the rest of this article, was discretized in order to form a few large classes and then used as a feature set.

In post hoc experiments, we have examined further features. The following two of them improved classification performance:

**Bigram features (B)** Bigrams of stemmed words are taken as features if they appear at least 3 times in the training material. E.g., the two most frequent bigrams with “interact” as first element are “interact with” (252 occurrences), “interact the” (53 occurrences).

**Syntactic features (S)** Our numeric PPIscore (feature P as described above) integrates different subscores (for example syntactic path, word at the top of the path, protein pair salience, etc.) into a single numeric value. For maximum entropy optimization, whose strength consists in weighting many different and even dependent pieces of information, this is not optimal. Therefore, we extracted and discretized more fine-grained numeric features from the PPI pipeline. To account for text zoning effects, this was done separately for the article titles (*TITLE*) and the abstract text (*TEXT*): *SYNREL* is a feature that counts how often an interaction-related dependency configuration between two proteins was found. *SURFACE* counts how often an interaction word and two proteins co-occur within a certain chunk distance. This feature is a back-off of the *SYNREL* feature. *SAMECHUNK* counts how often two proteins co-occur in the same chunk. *COOCC* is a new feature especially added for the PPI-ACT task that counts how many proteinss co-occur within a sentence.

Interestingly, the strongest features in this group, and overall the strongest features for class 0 (i.e. articles which do not contain useful PPIs), are *TEXTSYNREL*=0 and *TITLESYNREL*=0, i.e. the fact that no interaction was found gives strongest evidence that an article does not deal with PPIs. Unfortunately, the converse is not true: high counts of *TEXTSYNREL* turned out to be relatively weak evidence for class 1 (i.e. curatable articles). One reason for this result may be the fact that our term recognizer originally pooled together proteins and genes. Although we modified it for the competition, the distinction remained weak, which in turn led to incorrect classification of protein-protein interactions versus other types of interactions (e.g protein-gene).

#### Probabilistic maximum entropy classification

In a text classification task like ACT, applying machine learning optimization is indispensable for state-of-the-art results of balanced evaluation measures such as F-Score, Matthews correlation coefficient (MCC), or the area under the curve of the interpolated precision/recall curve (AUC iP/R). For a definition of these measures please see [17]. For our experiments, we relied on the maximum entropy [18] classification tool megam [19] which is simple to use and easily handles feature sets with hundreds of thousands of features. The conjugate gradient algorithm of megam takes only a few seconds on modern hardware when working with the development set and all features.

Another crucial advantage of megam is the fact that it basically computes a probabilistic classification decision with values between 0 and 1 according to the optimal weights of all features. For binary classification, a decision rule with a discretization threshold (henceforth abbreviated as DTH) is applied. The standard rule works as follows:

• *class* = 1 if *value* ≥ 0.5

• *class* = 0 if *value* < 0.5

Therefore, if we want to boost class 1 we can simply set the DTH lower than 0.5. For run 2, we set DTH at 0.2 which was determined by cross-validation on the development set. Further, the numerical distance between the value and the DTH gives us a confidence score as required for the ACT task. For AUC iP/R, only the relative confidence score ranking inside each class is relevant, consequently the following rule implements our scoring:

• *confidence* = *value* if *class* = 1

• *confidence* = 1 – *value* if *class* = 0

### Detection of experimental methods (PPI-IMT)

We have used two systems for our participation in the Biocreative III challenge, which we will refer to as system A and system B in the rest of this paper. While system A has been specifically optimized for the IMT task with task-specific heuristics, system B provides a fairly generic implementation of a naive Bayes multiclass classifier, which therefore does not need a very detailed description. In the rest of this section we provide more information about System A.

For all submitted results and experiments, we have obtained all statistical information from the training corpus, except that we adjusted the prior probability *p*(*method*) taking into account the results obtained by testing on the development corpus. More explicitly, methods which achieved low precision were given a lower weight. For most experiments, except where indicated, we have used only the method section of the articles, which we detected by a series of manually written regular expressions.

#### Dictionary-based approach

As a first approach to the detection experimental methods, a standard approach to named-entity recognition, e.g. finite state pattern matching using a dictionary resource can be used [20]. A dictionary of experimental methods, from the PSI-MI ontology, was distributed by the organizers in the shared task. However, only 10-15% of the methods sections of all PPI-IMT subtask articles contain matches to full PSI-MI terms or synonyms to terms, which immediately indicates that such an approach will necessarily have very low recall. The percentage of matches in the documents is an upper bound to a simple approach based on this method.

The vast majority of PSI-MI method terms and synonyms are multi-word terms, for example MI:0004 has the term *affinity chromatography technology* and the synonyms *affinity chrom* and affinity purification. Subsets or recombinations of these strings are often used in research articles instead of the official term or a given synonym. We therefore use submatches at the word level. For instance, an occurrence of the word purification in the methods section of an article marks it as a candidate for MI:0004. About 80% of the methods sections of all PPI-IMT subtask articles contain word submatches, which indicates a much higher upper bound. On average, a document contains 24 submatches to any of the methods that have been assigned to it by the curators.

As a first purely dictionary-based approach, we have thus used a pattern matcher giving high scores to every occurrence of an exact match to a method name and lower scores to every occurrence of a submatch. No stop word list has been used, except for filtering the prepositions *of* and *in*, which occur in many terms and synonyms. The inclusion of submatches can be expected to overgenerate, in other words to considerably increase recall, while precision will be low. As an intermediate level between full match and word-based submatch, we have used a subset approach: if more than three words of a term or a synonym from the PSI-MI dictionary appear in a ten word observation window, a mid-range score is given for each occurrence. The results that we have obtained are presented in the *“Results and Discussion”* section.

#### Dictionary-based approach with term probabilities from the corpus

The submatch-based approach just described delivers reasonable recall, but precision is low, and does not increase very much when setting high thresholds. In order to improve the system, it is advisable to learn from the corpus, and thus to abandon purely dictionary-based approaches. We have observed that, on the one hand, some submatch words are contained in many different experimental methods (they do not discriminate well), and on the other hand, that many submatch words very often do not refer to a method. For example, MI:0231 has the term name *mammalian protein protein interaction trap*, which means that every occurrence of the word *protein* assigns a score to this method.

To respond to these observations, a statistical method can be used. We use, on the one hand, conditional probabilities for the method given a word *p*(*method|word*) and, on the other hand, the probability of a word to be a term, its “method termness”. The method termness probability is measured as the conditional probability that a word occurence is actually part of one of the method terms of the document, i.e. where the annotator has assigned a method that contains the word to the document *p*(*termword* = *yes|word*, *document*)*.* For example, 83% of the occurences of the word “anti” come from documents where methods containing “anti” (e.g. *“anti bait coip”*) have been used.

An excerpt of method termness probabilites is given in table 1, left hand side.

**Table 1:** Termness and *p*(*method*|*word*)

Method termness		*p*(*method*|*word*)		
Probability	term word	Probability	word	method

0.831498470948	anti	0.490056818	L1	MI:0006
0.692307692307692	pooling	0.47027027	LT	MI:0019
0.662971175166297	hybrid	0.447269303	ERK1/2	MI:0006
0.519792083166733	x-ray	0.443877551	hydrogen-bonding	MI:0114
0.515198153135822	coimmunoprecipitation	0.441441441	omit	MI:0114
0.484276729559748	coip	0.43876567	synapses	MI:0006
0.469194312796209	bret	0.436363636	tumours	MI:0006
0.396292409933543	fret	0.435114504	REFMAC	MI:0114
0.369761273209549	tag	0.430695698	p21	MI:0006
0.367924528301887	tomography	0.424657534	COOT	MI:0114
0.35606936416185	bifc	0.423558897	epithelium	MI:0006
0.35405192761605	diffraction	0.418918919	flower	MI:0018
0.329399141630901	resonance	0.417443409	IKK	MI:0006
0.322784810126582	epr	0.412797992	caspase-3	MI:0006
0.322607959356478	crystallography	0.407843137	NF-kB	MI:0006
0.312878528168209	two-hybrid	0.406961178	floral	MI:0018
0.311203319502075	2-hybrid	0.406926407	9.00E+10	MI:0007
0.307599517490953	itc	0.404040404	diffracted	MI:0114
0.307372793354102	spr	0.40311174	atom	MI:0114
0.303317535545024	biosensor	0.403057679	HIV-1	MI:0007
0.300881858902576	two	0.40167364	wwwpdborg	MI:0114
0.300359712230216	saxs	0.401408451	CCP4	MI:0114
0.296829971181556	bimolecular	0.39668175	BK	MI:0006
0.296758104738155	plasmon	0.39629241	FRET	MI:0055
0.283073367995378	bait	0.394624313	MCF-7	MI:0006
0.282754418037782	fluorescence	0.394136808	contoured	MI:0114
0.282689623080503	nmr	0.39047619	Å	MI:0114
0.272583201267829	isothermal	0.389684814	hypoxia	MI:0006
0.258223684210526	calorimetry	0.387915408	c-Myc	MI:0007
0.258064516129032	one-hybrid	0.387096774	PI3K	MI:0006
0.247863247863248	crosslink	0.385964912	specification	MI:0018
0.238479262672811	tap	0.385809313	seed	MI:0018
0.222466960352423	phage	0.38559322	15N	MI:0077
0.21827744904668	scattering	0.384858044	colorectal	MI:0006
0.214154411764706	pull	0.384114583	Å2	MI:0114
0.211344922232388	force	0.38247012	carboxylate	MI:0114
0.205298013245033	bn-page	0.38225925	Src	MI:0006
0.203338930508912	yeast	0.381818182	Argonne	MI:0114
0.181818181818182	bioluminescence	0.38125	Floral	MI:0018
0.174830377336031	kinase	0.380802518	Mdm2	MI:0006
0.174038675261169	down	0.380634391	carbonyl	MI:0114

The score for each method of a document is the summation of its values:

 for a document containing *n* word tokens. We use this statistical model for all words that are matches or submatches of the terms given in the PSI-MI dictionary. As we see in the *“Results and Discussion”* section, performance increases considerably.

#### Corpus-driven approach

As a statistical data-driven model for terms increases the performance, one may wonder if the same approach can also be used for words that are not terms. To test this, we discarded the term dictionary, learned the score for each method (as described above) for all words from the training resource and apply it to all words, whenever *p*(*method|word*) and *termness*(*word*) are above 10%, and whenever the word is used in at least 5 training documents.

The lists containing words with high probabilities are not obviously interpretable to the non-expert, although some of the inherent knowledge they contain are clear hints. An excerpt of frequent words indicating experimental methods at high probability is given in table 1, right hand side.

#### Combinations

Better results can be obtained by combining the corpus-driven and the dictionary-based method. We have simply added up the scores of each of the approaches. For one submission to the shared task (run 5), we have also used a version that averaged the scores of system A and system B.

#### Post-hoc experiments on run 1

After the shared task, we have further improved the model. First we have added a bigram model, *p*(*method*|*bigram*), again thresholding at 10 percent. This led to a very strong increase in performance. [21] has shown that bigrams can be used very effectively in a word sense disambiguation task. In order to weight multi-word terms more strongly, we have added a collocation measure weight (the chi-square score [22] in our case).

## Results and discussion

### Article classification task (PPI-ACT)

Table 2 shows our official results of the shared task and gives an overview of the features and techniques used for the different runs. In our participation, we have submitted three runs applying Maximum Entropy (ME) optimization. The feature weights used for the test set were drawn from the development set only. The inclusion of the balanced (but therefore biased) training set (which was released earlier in the shared task) proved to deteriorate the results in a 10-fold cross-validation experiment on the development set. According to our results, the development set proved to be representative for the test set. Run 3 and run 4 used only the result of the PPI pipeline (as developed for the BioCreative II.5 shared task) for comparison. Run 1 was aimed at maximizing accuracy. Specificity was deliberately maximized at the cost of sensitivity because of the class imbalance.

**Table 2:** Results and properties of our official runs.

Run	Acc	Spec	Sens	F-Score	MCC	AUC iP/R	ME	Feat	DTH
Official run 1	88.68	97.64	38.57	50.83	0.48297	63.85	+	WMP	0.50
Official run 2	87.93	93.06	59.23	59.82	0.52727	63.89	+	WMP	0.20
Official run 3	67.05	64.19	83.08	43.34	0.34244	41.74	–	P	
Official run 4	73.68	74.13	71.21	45.08	0.34650	41.74	–	P	
Official run 5	88.00	94.40	52.20	56.89	0.50255	62.39	+	WM	0.25

Post hoc run 2a	86.90	90.57	66.37	60.58	0.53089	64.06	+	WMP	0.20
Post hoc run 6	87.53	91.57	64.95	61.24	0.53969	66.30	+	WMPBS	0.21

Additionally we give our post hoc run 2a, performed without a minimal feature count threshold of 3 and bi-normal feature selection (20,000). Our best post hoc run 6 uses bigrams (B) and syntactic features (S). Features considered are W (bag of words), M (MeSH), P (PPIscore), B (bigrams), S (syntactic) - for a detailed description see page 3.

Run 2 was aimed at maximizing Matthews correlation coefficient (MCC) by applying a discretization threshold (DTH) of 0.2 (see the methods section on PPI-ACT for an explanation of the discretization threshold (page 5). The DTH of 0.2 was determined empirically on the basis of the development set. MCC (also known as phi coefficient) is regarded as a balanced evaluation measure for binary classification prediction problems with an imbalanced class distribution [23]. A value of 0 represents an average random prediction and a value of 1 means perfect prediction. Its formal definition in terms of true/false positives/negatives is as follows:

As expected, this run results in the best MCC, and it is at the same time our run with the highest AUC. Run 3 was aimed at high sensitivity using the raw numeric PPIscore (without applying ME optimization). We used the following decision rule: if *PPIscore* > 0.2 then *class* = 1 else class = 0. The low results of this run show that the raw PPIscore alone is not specific enough and produces too many false positives. Run 4 was aimed at a balanced specificity and sensitivity using the raw PPIscore (without applying ME optimization). We adapted the decision rule of run 3 as follows: if *PPIscore* > 1.1 then *class* = 1 else class = 0. Although we did not reach equal specificity and sensitivity on the test set, F-Score and MCC improved with respect to run 3.

Run 5 was restricted to bag-of-words (W) and MeSH (M) features and was aimed at assessing the performance of easily available text classification features. We applied a DTH of 0.25 in order to maximize MCC (threshold derived from the development set). Although this run represents a baseline methodically, the results are quite high. The comparison with our best run 2 is particularly interesting because it shows the impact of the PPIscore feature: we gain 64 true positives, but also get 68 more false positives.

#### Post hoc experiments for run 2

In the official run 2 we decided to use only features appearing at least 3 times in the development set. Additionally, we used the fast bi-normal features selection threshold of megam with a value of 20,000 [24]. These thresholds were determined on the basis of cross-validation runs on the development set using the criterion of overall accuracy. However, overall accuracy proved to be an unfortunate choice, because applying these globally appropriate thresholds hampered the recognition quality of the smaller class 1 (i.e. articles containing PPIs).

In order to quantify the negative effect, we retrained the classifier of run 2 with the same development set data features without any threshold and applied them to the test set. Table 2 contains the resulting numbers of this run 2a in comparison to the official results for run 2. As expected, the overall accuracy is lower now, whereas MCC (which was the optimization measure of our run 2) improves considerably: in comparison with all officially evaluated results, it would bring up this particular run from rank 11 to rank 5.

#### Dealing with the class imbalance problem: Classification threshold lowering vs. oversampling

[25] discuss the problem of skewed class distributions for text classification. They also mention different methods of resampling the data into balanced partitions in order to fix the typical under-representation of minority classes in the results. In their experiments for the BioCreative II.5 article classification task, downsampling the negative examples was inferior to oversampling positive examples. The simple and effective technique of oversampling using duplicates proved to be sufficient in their case.

For run 2 and 5 we used the technique of DTH lowering, i.e. we changed the classification decision rule of our probabilistic maximum entropy classifier. To assess the quality of our technique with respect to simple oversampling, we conducted the following 10-fold cross-validation experiment on the development set: each cross-validation training set had originally 612 positive (17%) and 2979 negative examples (83%). In order to attain balanced classes with 2979 positive examples, each positive example was taken 4 times and additionally 531 positive examples were chosen randomly a 5th time.

Table 3 shows the results for the mean values in a stratified 10-fold cross-validation experiment on the development set using oversampling with a standard DTH of 0.5 and DTH lowering (0.20) using the features of run 2. No restrictions on minimal feature occurrence or feature set size were used in the maximum entropy classifier training. According to these results, oversampling is inferior to classification threshold lowering with respect to almost all evaluation measures. In particular, AUC iP/R suffers substantially from oversampling.

**Table 3:** Comparison of mean results of a stratified 10-fold cross-validation experiment on the development set using oversampling versus using discretization threshold (DTH) lowering.

Method	Acc	Spec	Sens	F-Score	MCC	AUC iP/R	Feat	DTH
Oversampling	86.72	92.22	59.93	60.49	0.52580	66.36	WMP	0.50
DTH lowering	85.94	89.76	67.35	61.92	0.53689	69.33	WMP	0.20
DTH lowering	87.84	93.81	58.82	62.21	0.55363	69.33	WMP	0.36

No restrictions on minimal feature occurrence or feature set size were used in these experiments, therefore the DTH of 0.20 is no longer optimal. A DTH of 0.36 gives the best results overall. Features considered are W (bag of words), M (MeSH), P (PPIscore), B (bigrams), S (syntactic) - for a detailed description see page 3.

#### Assessment and comparison of the optimal performance of different feature sets

In order to gain more insights on the performance of the different features sets used in our runs, we conducted a systematic feature ablation experiment on the development set. In a stratified 10-fold cross validation (CV) experiment, the maximal performance of each feature set and of their combinations was assessed.

**Figure 1:**
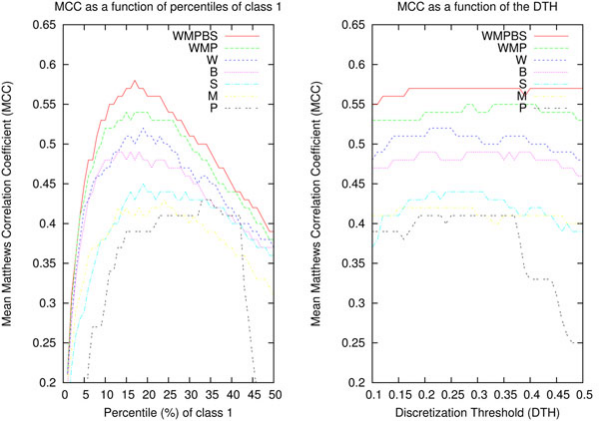
Plots of mean MCC on the development set in a stratified 10-fold cross validation experiment with different feature sets. In the left panel mean MCC is a function of the percentile of class 1 decisions. In the right panel mean MCC is a function of varying DTHs.

We chose MCC as a consistent optimization criterion because of its known advantages when evaluating imbalanced classes. However, the use of a fixed DTH, e.g. 0.2 as for run 2, is not appropriate for comparing feature sets with very different characteristics as for instance bag-of-words and PPIscore. To compute the optimal thresholds for a feature set in a CV setting, one can systematically test different thresholds and select the best performing on average. The plot on the right of Fig. 1 shows the mean MCC as a function of different DTHs for the feature sets used in our experiments. As can be seen easily from the plot, the better the feature set is, the less important is the choice of the exact DTH. However, for weaker feature sets it is difficult to determine the optimal DTH for maximal MCC.

When applying fixed DTHs, the number of items classified as class 1 (documents categorized as containing a PPI) varies slightly for each CV test subset. Another solution is to ensure that every CV test subset contains the same percentile of items classified as class 1. In this case, the DTH varies slightly for each CV test subset. The plot on the left of Fig. 1 shows the mean MCC as a function of different percentiles of class 1 classifications. As can be seen easily from this plot, better feature sets converge to the percentile of the real distribution of class 1 in the development set (17%). Unlike the plot for fixed DTHs, also weaker feature sets clearly show optimal percentiles for maximal MCC. Therefore, we compared the maximal performance of the different feature sets based on their best performing percentiles.

In order to statistically compare the optimal performance of different features sets in terms of MCC, we applied the t-test for dependent samples pairwise between the 10 test subsets of our CV experiments (*df* = 9). With this methodology we can answer the following questions:

• *p* – *value*: Does feature set *f*_1_ perform significantly (5% level, i.e. *p* – *value* < 0.05) better than feature set *f*_2_?

• EI: How big is the minimally expected improvement (95% confidence) of this difference between *f*_1_ and *f*_2_?

• CI: What is the confidence interval (95%) of feature *f*_1_ rsp. feature *f*_2_ ?

We used R’s implementation of the t-test to calculate these statistics (applying the Shapiro-Wilks test of normality beforehand). The calculation of the estimated improvement are based on separately estimated variance for each feature set and the Welch modification to the degrees of freedom is used. Table 4 contains the most important findings for each feature set and their combinations. Although the feature set combination WMPBS at percentile 17 was not significantly better than WMS or WMBS, it produced the best run on the test set (see Table 2). With these results, we would reach rank 3 for MCC and AUC iP/R when compared with the official results of the competition.

**Table 4:** Comparison of feature set quality using t-test on stratified 10-fold CV subsets from the development set at best performing percentiles (per) of class 1.

*f*_1_ @ per	CI	*f*_2_ @ per	CI	p	EI
S @ 19	0.415, 0.480	P @ 35	0.397, 0.454	0.0379	0.022
B @ 13	0.460, 0.511	M @ 13	0.370, 0.461	0.0082	0.070
B @ 13	0.460, 0.511	S @ 19	0.415, 0.480	0.0273	0.038
W @ 20	0.491, 0.535	B @ 13	0.460, 0.511	0.0116	0.028
WS @ 22	0.510, 0.568	W @ 20	0.491, 0.535	0.0043	0.026
WMS @ 20	0.546, 0.577	WS @ 22	0.510, 0.568	0.0414	0.023
WMBS @ 17	0.549, 0.586	WS @ 22	0.510, 0.568	0.0179	0.029
WMPBS @ 17	0.558, 0.595	WS @ 22	0.510, 0.568	0.0122	0.038

Features considered are W (bag of words), M (MeSH), P (PPIscore), B (bigrams), S (syntactic) - for a detailed description see page 3. Interpret the rows as follows (e.g. row 4): Feature set W has a 95% confidence interval (CI) of [0.491, 0.535], feature set B has one of [0.460, 0.511]. According to a t-test for dependent samples, feature set W is significantly better than feature set B (df=9; p=0.0116). The expected improvement (EI) of the MCC measure is at least 0.028 (95% confidence level). Notice, that feature set PBMSW or BMSW are not significantly better than MSW. For the case of combinations of 2, 3 or 4 different feature sets, only the best performing ones were selected in this table.

### Detection of experimental methods (IMT)

For our participation in the shared task, we have developed two statistical systems, which in this document are referred to as system A (runs 1 and 3) and system B (runs 2 and 4). Both are based on a naive Bayes approach but use different optimizations and heuristics. We mainly describe system A here.

#### Dictionary-based approach

Our pure dictionary-based approach has been described in the *“Methods”* section. Using thresholds on the scores it is possible to increase precision at the cost of recall. For the dictionary-based approach we obtain the results given in table 5 column 1 (*TermDict*).

**Table 5:** Performance Development of PPI-IMT system A

IMT	1. TermDict	2. TermDict p(m|term)	3. Word Corp	4. run 1 submit	5. Bigram p(m|bi)	6. colloc chi^2^	7. without zoning	8. comb. run 2
Evaluated Res.	4347	4334	2355	5098	11103	11094	15749	21600
TP	417	417	369	447	486	486	522	527
FP	3930	3917	1986	4651	10617	10608	15227	21073
FN	110	110	158	80	41	41	5	0

Micro P	0.09593	0.09622	0.15669	0.08768	0.04377	0.04381	0.03314	0.02440
Micro R	0.79127	0.79127	0.70019	0.84820	0.92220	0.92220	0.99051	1.00000
Micro F	0.17111	0.17157	0.25607	0.15893	0.08358	0.08364	0.06414	0.04763
Micro AUC iP/R	0.21694	0.26532	0.21633	0.27588	0.29466	0.29712	0.30205	0.30034

Macro P	0.10308	0.10333	0.16587	0.09346	0.04532	0.4537	0.03312	0.02440
Macro R	0.77590	0.77590	0.69459	0.83206	0.91261	0.91261	0.99174	1.00000
Macro F	0.17502	0.17542	0.25564	0.16322	0.08517	0.08525	0.06359	0.04735
Macro AUC iP/R	0.40387	0.46438	0.39722	0.47884	0.50159	0.50336	0.50630	0.50890

#### Dictionary-based approach with term probabilities from the corpus

Our dictionary-based approach which learns term probabilities from the corpus has also been described in the *“Methods”* section. The performance is given in table 5, column 2. It is interesting to notice that precision and recall of the dictionary-based approach (column 1) and the unfiltered output (column 2) are nearly identical, however the latter has a better AUC. Therefore, if one filters both results using a threshold based on score or rank, in the second case higher precision and recall will be achieved.

#### Corpus-driven approach

Our corpus-driven approach has been described in the methods section. Even without any dictionary resource, the approach performs surprisingly well, as table 5, column 3 shows. In terms of AUC iP/R, the performance is slightly lower than the dictionary-based approach with term probabilities from the corpus (column 2), but very similar to the purely dictionary-based approach (column 1). In terms of F-score it is clearly better. This indicates that corpus-driven document classification approaches may in general be a suitable approach to experimental methods detection, particularly in the given setting: in the training corpus, the experimental methods associated to each document are given, but there is no information on the position or the string expressing the use of the method.

Recent named-entity recognition approaches [26,27] use string-similarity scorers that learn from the dictionary or from a training corpus. Both significantly improve performance, the latter leads to even better results. The corpus-driven approach used in this version is more radical than the promising approaches of [26,27], instead of extending an existing dictionary-resource, it does not use any dictionary but learns method discriminators from the training corpus as if it were a supervised text-classification task. [10] use hand-crafted dictionary patterns to respond to the observation that existing experimental methods dictionaries have very low coverage. We go beyond this by learning the discriminators from the training corpus.

After the submission to the shared task, we have added the following improvements. Adding a word bigram model *p*(*method*|*bigram*) increased the performance considerably, as table 5, column 5 shows. We have further tested if weighting bigrams by their collocation weight increases performance. Word combinations with high collocation weight are often terms, collocation measures can be used for term detection. Thus, table 5, column 6 shows a small improvement. Finally, we have tested how the performance changes if we use the entire articles instead of only the method subsection. Surprisingly, using the entire article also leads to a small increase, particularly increasing recall.

The marginally best performance is obtained by combining system A and system B, as table 5, column 8 shows. We have conducted some additional experiments, always leading to similar or slightly worse performance. We believe that we are getting close to the ceiling of our approach.

Table 6 compares the individual experimental methods (of the system given in table 5, column 6). Precision and recall of the full output is given on the left, while only results above a threshold are given on the right. The comparison shows that while most methods, also slightly rarer methods, attain good recall, the ranking of rare methods is consistently too low. However, increasing the weights of rare methods always led to a worse overall performance. Particularly good performance has been observed on a few methods, which in some cases (e.g. MI:0416) might be due to additional terminology which was added after manual inspection of the training data, while in others (e.g. MI:0405, MI:0029) usage of bigrams and collocations may have helped boosting our results.

**Table 6:** Performance by experimental method, for all methods where f(train) > 20

	Frequencies			Full Output		Thresholded	
	f(train)	f(develop)	f(test)	P	R	P	R

ALL	4348	1379	527	4.38%	92.22%	15.70%	80.46%

MI:0006	736	246	60	27.03%	100%	27.03%	100%
MI:0007	728	212	66	29.73%	100%	29.73%	100%
MI:0096	438	198	98	44.14%	100%	44.14%	100%
MI:0018	403	85	30	13.51%	100%	13.51%	100%
MI:0114	223	50	13	5.86%	100%	6.25%	100%
MI:0416	172	83	61	27.48%	100%	27.59%	91.80%
MI:0071	180	35	13	5.86%	100%	6.84%	100%
MI:0424	416	44	15	6.76%	100%	7.41%	93.33%
MI:0107	82	19	19	8.80%	100%	23.68%	94.74%
MI:0663	68	35	2	0.93%	100%	0%	0%
MI:0065	61	16	6	2.45%	83.33%	0%	0%
MI:0077	58	11	7	3.33%	100%	20.69%	85.71%
MI:0028	51	9	1	0.47%	100%	0%	0%
MI:0030	46	20	2	0.98%	100%	0%	0%
MI:0676	45	15	8	3.69%	100%	16.67%	100%
MI:0055	45	9	11	5.31%	100%	32.14%	81.81%
MI:0809	41	14	0				
MI:0415	40	22	2	1.04%	100%	0%	0%
MI:0004	35	9	6	3.03%	100%	0%	0%
MI:0029	34	11	6	3.16%	100%	0%	0%
MI:0040	31	9	0				
MI:0404	30	12	1	0.49%	100%	0%	0%
MI:0051	29	9	2	1.10%	100%	0%	0%
MI:0017	29	7	0				
MI:0808	28	3	1	0.48%	100%	0%	0%
MI:0047	28	12	5	2.50%	100%	0%	0%
MI:0405	27	4	7	3.55%	85.71%	0%	0%
MI:0049	27	13	1	0.47%	100%	0%	0%
MI:0019	24	3	51	37.25%	100%	0%	0%
MI:0410	23	0	0				
MI:0413	21	13	0				

#### Submission to the shared task

Combining the corpus-driven approach and the dictionary-based approach that learns term probabilities from the corpus can be expected to perform better than the individual approaches. This is indeed the case, as table 5, column 4 (*run1 submit*), illustrates. This is the system that we have submitted as run1 to the shared task. Table 7 reports the official results obtained in the competition, where run 1 and 2 are the full outputs of system A and system B (respectively), while run 3 and 4 are optimized outputs of the same systems (obtained by setting a rank threshold). Run 5 is the combined output of both systems, obtained averaging the scores of run 1 and 2 for each method.

**Table 7:** PPI-IMT Performance of the submitted runs

IMT	run 1 (A)	run 2 (B)	run 3 (A)	run 4 (B)	run 5 (A+B)
Evaluated Results	5098	21529	4576	666	21600
TP	447	527	431	223	527
FP	4651	21002	4145	443	21073
FN	80	0	96	304	0

Micro P	0.08768	0.02448	0.09419	0.33483	0.02440
Micro R	0.84820	1.00000	0.81784	0.42315	1.00000
Micro F	0.15893	0.04779	0.16892	0.37385	0.04763
Micro AUC iP/R	0.27588	0.24484	0.27727	0.14169	0.29016

Macro P	0.09346	0.02448	0.09992	0.33483	0.02440
Macro R	0.83206	1.00000	0.79377	0.42883	1.00000
Macro F	0.16322	0.04750	0.17163	0.35403	0.04735
Macro AUC iP/R	0.47884	0.44034	0.47650	0.30927	0.50111

The full outputs were aimed at maximizing recall and AUC, the optimized outputs at increasing F-score. We have avoided sending runs which optimize precision, because these can always be obtained by selecting for each article only the best prediction, i.e. the method which is ranked first. [6] reports that the curators preferred a high recall setting to a high precision setting, because it is much easier and less time-consuming to reject suggestions (false positives, low precision) than to add new information from scratch (false negatives, low recall). We believe that a good ranking, coupled with good recall, offers the optimal setting in order to allow the user to decide where to stop examining the results, rather than leaving the decision to the system.

It is interesting to notice that run 5 achieves a relatively high AUC (0.5011), only marginally lower than the best reported AUC (0.5297) in the official competition. However, our submission retains full recall (like run 2), while the submission which achieved best AUC had a recall of 59.90%.

From these results we can conclude that system A produces a better ranking, which, when combined with the more complete output of system B, results in a better AUC.

## Conclusions and outlook

In this paper we described effective solutions to two crucial problems in the curation of protein-protein interactions from the literature: determining which articles should be curated, and extracting support information from the articles (in particular which experimental methods have been used to detect the interactions).

Solutions to the former problem (text classification) have in the past relied on classical machine learning techniques. Recent advances in natural language processing are now brought to bear on this problem, for instance the use of high-quality syntactic parses for improved PPI detection [28,29], offering novel ways to approach this task. Our own results, together with those of other participants, clearly show that natural language processing tools are now part and parcel of the set of techniques that advanced text mining systems can rely upon. The view that using PPI features for the PPI-ACT task is useful is supported for example by [30], who have added interaction trigger keywords (e.g. activate, down-regulate, etc.) and sentence-level co-occurrence of protein names to a bag-of-words PPI-ACT approach, achieving best results in the ACT task of BioCreative II. Moreover, the system which achieved the best results in the PPI-ACT task of BioCreative III [31] exploited a rich set of features derived from a dependency parser, which contributed significantly to their achievement.

As for the latter problem (detection of the experimental methods), the results presented in this paper clearly show that a problem originally assumed to be a type of named entity recognition can be successfully approached as a text classification task. Nevertheless, best results are obtained by combining different information sources. In particular, we have used task-blind lexical associations (derived from the training data) between words and methods, as well as task-aware relevance of given terminology (fragments of method names), as provided in official resources.

In future work we would like to improve our results in these two tasks by exploiting more training material (such as the resources provided in previous BioCreative competitions, as well as experimenting with different learning techniques. For example, [32] show that adding unlabelled and weakly-labelled data to the training set significantly improves results on an SVM-based approach.

Although current results already reach usability level, further improvements are possible, which, together with satisfactory user interfaces, would certainly help to increase the acceptance of text mining tools within the biocuration community and beyond.

## List of Abbreviations

In order of appearance in the text, the following abbreviations were used:

PPI: Protein-protein interaction; ML: Machine learning; ACT: Article classification task (a Biocreative III task); W: Bag-of-words features; M: MeSH features; P: PPI Score features; B: Bigram features; S: Syntactic features; MCC: Matthew’s correlation coefficient; AUC iP/R: Area under curve of the interpolated precision/recall curve; DTH: Discretization threshold; IMT: Interaction method task (a Biocreative III task); ME: Maximum entropy; CV: Cross-validation; EI: Expected improvement; CI: Confidence interval

## Authors’ contributions

GS implemented most of the IMT system, provided the syntactic features used in the PPI-ACT system, and is the main author of the corresponding sections of this paper. SC implemented the PPI-ACT system and is the main author of the corresponding sections of this paper. FR supervised the activities of the group, implemented part of the IMT system and authored various parts of this paper. All authors read and approved the paper.

## Competing interests

The authors declare no competing interests.
